# *Hibiscus moscheutos* L. Flower Petals Extract Phenolic Profile and In Vitro Antimicrobial, Biofilm Formation, Autoaggregation, Prebiotic, Genotoxicity, and Anti-Inflammatory Properties

**DOI:** 10.3390/molecules30173569

**Published:** 2025-08-31

**Authors:** Patryk Kowalczyk, Elżbieta Klewicka, Joanna Milala, Lidia Piekarska-Radzik, Elżbieta Karlińska, Michał Sójka, Robert Klewicki

**Affiliations:** 1 Institute of Fermentation Technology and Microbiology, Faculty of Biotechnology and Food Sciences, Lodz University of Technology, 171/173 Wólczańska St., 90-530 Lodz, Poland; lidia.piekarska-radzik@p.lodz.pl; 2 Institute of Food Technology and Analysis, Faculty of Biotechnology and Food Sciences, Lodz University of Technology, 4/10 Stefanowskiego St., 90-924 Lodz, Poland; joanna.milala@p.lodz.pl (J.M.); elzbieta.karlinska@p.lodz.pl (E.K.); michal.sojka@p.lodz.pl (M.S.); robert.klewicki@p.lodz.pl (R.K.)

**Keywords:** *Hibiscus moscheutos*, plant extract, polyphenols, antimicrobial activity, biofilm formation, autoaggregation, prebiotic activity, genotoxicity, anti-inflammatory activity

## Abstract

The flowers of *Hibiscus moscheutos* L. are among the largest within the genus, and the plant contains numerous nutrients and phytochemicals that perform various structural and regulatory functions in the human body upon consumption. However, these properties remain insufficiently explored. In this study, the phenolic composition and in vitro biological activity of an ethanolic extract from *H. moscheutos* petals were investigated. The total phenolic content was 219.52 mg/g (HPLC method), including phenolic acids (5.17 mg/g), flavanols (59.18 mg/g), flavonols (93.09 mg/g), and anthocyanins (62.08 mg/g). Many species of the genus *Staphylococcus*, as well as two probiotic strains of *Lacticaseibacillus* spp., were sensitive to the extract’s effects (100 mg/mL), which appeared to be strain-dependent. The MIC values for *Staphylococcus* spp. ranged from 6.25 to 100.00 mg/mL, while for the two probiotic strains, they were 12.50 and 100.00 mg/mL, respectively. The extract did not show prebiotic activity. Nevertheless, it enhanced the biofilm-forming ability of both probiotic and pathogenic microbiota on abiotic (polystyrene) and biotic (mucin and gelatin) surfaces. The stimulation of *Staphylococcus* spp. biofilms is considered undesirable and may justify limiting the use of the extract, for example, in pharmaceutical or medical applications. At concentrations above 25 mg/mL, the extract reduced bacterial autoaggregation. It also exhibited low genotoxicity in the Ames test and demonstrated anti-inflammatory activity comparable to sodium diclofenac. *Hibiscus* petal extracts might represent a promising source of bioactive compounds for novel pharmaceutical, nutraceutical, and food applications; however, their potential requires further in-depth investigation.

## 1. Introduction

Over the millennia, traditional medicine based on the use of medicinal plants has played a crucial role in alleviating symptoms and treating a wide range of human diseases and ailments. The oldest written record of herbal medicine use was found on a Sumerian clay tablet from Nagpur, India, dating back approximately 5000 years [1,2]. Today, herbal remedies continue to be an important part of folk traditions among rural communities in developing countries [3].

According to reports by the World Health Organization (WHO), more than 80% of the global population, particularly in low- and middle-income countries, relies on traditional medicine as a primary form of healthcare [3,4]. Moreover, a significant proportion of active compounds used in modern pharmacotherapy, at least 25%, are either of plant origin or represent structural analogs of phytochemicals originally isolated from medicinal plants [3]. In recent decades, there has been a significant increase in interest in natural pharmaceuticals, driven by the declining effectiveness of synthetic drugs and the growing number of contraindications associated with their use [1,4]. Additionally, plants are considered attractive pharmaceutical raw materials due to their low cost, reduced risk of side effects compared to synthetic drugs, very low toxicity, and the potential for organic production [3].

Medicinal plants contain a wide range of biologically active compounds, typically synthesized through secondary metabolism, such as flavonoids, alkaloids, terpenoids, and tannins, which exhibit significant effects on the human body. Plant extracts are considered a promising source of new drugs, as demonstrated in previously published studies showing their effectiveness in the treatment of many modern diseases [5]. Due to their antimicrobial properties, some of these extracts may be particularly useful against infections caused by microorganisms resistant to most well-known antibiotics. It is widely known that the therapeutic properties of plants do not result from the action of a single active compound but rather from the synergistic effect of multiple constituents. This synergy reduces the likelihood of resistance development and enables plant-based preparations to be highly effective against pathogens that are difficult to eliminate with a single synthetic drug [3,5]. This has encouraged researchers to conduct extensive investigations of plants historically used for medicinal purposes, as well as to search for new species with potential health-promoting properties [6].

The genus *Hibiscus* (family *Malvaceae*) comprises approximately 250–300 species naturally occurring in tropical and subtropical regions worldwide, many of which are cultivated for their ornamental value. *Hibiscus* species are commonly herbs, shrubs, or small trees, known for their diverse and beneficial properties [4,7]. Numerous studies have shown that plants belonging to this genus contain a wide variety of phytochemicals and secondary metabolites exhibiting antibacterial, antifungal, antiparasitic, anti-inflammatory, wound-healing, antioxidant, anti-aging, anticancer, antihypertensive, hypoglycemic, and antifertility activity [3,4,7,8,9]. To date, the biological activity of a narrow group of species (such as *H. sabdariffa*, *H. rosa-sinensis*, and *H. syriacus*) has been extensively described, as they are the most popular, historically known, and economically important species, whereas the properties of many other *Hibiscus* species remain largely unexplored.

*Hibiscus moscheutos* L., also known as Rose Mallow, swamp hibiscus, or hardy hibiscus, is an herbaceous perennial native to wetlands along the eastern coast of North America. It grows in freshwater and brackish marshes and swamps, sending up several to many upright stems each year from a perennial rootstalk, reaching heights of 1–2 m. The flowers are large (ranging from 5 to 30 cm in diameter), with colors varying from white through pink and red to violet, often featuring a deeper-colored base. The plant blooms abundantly from midsummer through late autumn [10,11,12]. Since colonial times, many Rose Mallow cultivars have been grown for their ornamental value, primarily in the United States and Europe. To this day, its most important economic use remains as an ornamental plant. As a cultivated species, it performs best in good garden soil with moderate watering. While no widespread food use is known, the seeds of *H. moscheutos* are reported to have medicinal applications due to their cordial, stomachic, nervine, pungent, demulcent, and emollient properties [13].

Although the flower petals of *H. moscheutos* are among the largest in the genus, their polyphenol profile and potential medicinal and health-promoting properties remain insufficiently investigated. In this study, a qualitative and quantitative analysis of the polyphenol composition was performed, and the in vitro bioactivity of an extract from *H. moscheutos* petals was evaluated. Its antagonistic potential against pathogens, microorganisms associated with food production or contamination, and probiotic strains was assessed. The extract’s effects on biofilm formation and autoaggregation of probiotic strains and *Staphylococcus* spp. were examined. Additionally, prebiotic activity, genotoxicity, and anti-inflammatory properties were analyzed. Ethanol was used to extract bioactive compounds, minimizing the risk of toxicity and providing a foundation for further research on developing new plant-derived therapeutics.

## 2. Results

### 2.1. Phenolic Composition

Table 1 shows the compositional characteristics of the phenolic compounds present in the extract of hibiscus petals. The total content of phenolic compounds determined by the spectrophotometric method in the lyophilized extract, expressed as gallic acid equivalent, was nearly 216.39 mg/g. Similar results were obtained from the HPLC method. The sum of phenolic compounds determined by chromatographic methods was 219.52 mg/g.

Groups of compounds such as phenolic acids (5.17 mg/g), anthocyanins (62.08 mg/g), flavonols (93.09 mg/g), and flavanols (59.18 mg/g) were present in the extract.

### 2.2. Antibacterial Activity and Minimum Inhibitory Concentrations (MICs)

According to Table 2, the ethanol extract of *H. moscheutos* flower petals, at a concentration of 100 mg/mL, exhibited antagonistic activity against 10 out of 28 bacterial strains. All susceptible strains were Gram-positive. One strain exhibited high sensitivity (≥25 mm), six showed moderate sensitivity (15–24 mm), and three displayed low sensitivity (11–14 mm); this classification was adapted from Canlı et al. [14].

Among the probiotic strains, the extract inhibited the growth of two out of four *Lacticaseibacillus* strains. *L. paracasei* ŁOCK 0919 was weakly inhibited (13.0 mm), whereas *L. casei* ŁOCK 0900 exhibited moderate sensitivity (21.0 mm). The minimum inhibitory concentrations (MICs) for these strains were 100.0 and 12.5 mg/mL, respectively.

Against pathogenic bacteria, the extract exhibited antagonistic activity only against certain *Staphylococcus* strains. Overall, 8 out of 13 *Staphylococcus* strains were sensitive to the extract, mostly showing moderate susceptibility. The highest sensitivity was observed in *S. capitis* MW 776357 (31.3 mm; MIC 6.25 mg/mL). Additionally, the extract inhibited the growth of all four *S. aureus* strains, with inhibition zone diameters ranging from 12 to 17 mm (MICs ranging from 100.0 to 25.0 mg/mL). Other sensitive strains included *S. epidermidis* MW 040699, *S. haemolyticus* MW 040704, and *S. saprophyticus* MW 040701. Notably, the extract exhibited antibacterial activity against all *Staphylococcus* spp. strains originally isolated from human skin, except for *S. epidermidis* MW 040703.

The susceptibility of each strain to two different antibiotics was evaluated as a positive control. At a concentration of 100 mg/mL, the extract exhibited stronger antagonistic activity against *S. capitis* MW 776357 (*p* = 0.002) and *S. haemolyticus* MW 040704 (*p* = 0.008) compared to cefoxitin (30 µg). Furthermore, no statistically significant differences were observed between the activity of the extract and the antibiotics ampicillin (2 µg) against *L. casei* ŁOCK 0900 (*p* = 0.312) and *L. paracasei* ŁOCK 0919 (*p* = 0.698), cefoxitin against *S. aureus* ATCC 27734 (*p* = 0.106), *S. epidermidis* MW 040699 (*p* = 0.632), and *S. saprophyticus* MW 040701 (*p* = 0.219), as well as gentamicin (120 µg) against *L. casei* ŁOCK 0900 (*p* = 0.312). In all other cases, the antibacterial activity of the extract was significantly lower than that of the tested antibiotics.

### 2.3. Antifungal Activity

The ethanol extract from *H. moscheutos* petals at a concentration of 100 mg/mL showed no antifungal activity against any of the tested yeast or filamentous fungal strains. In the positive controls using nystatin (0.21% *w*/*v*), the inhibition zone diameters ranged from 22 to 33 mm.

### 2.4. Effect of the Extract on Biofilm Formation

**Figure 1:**
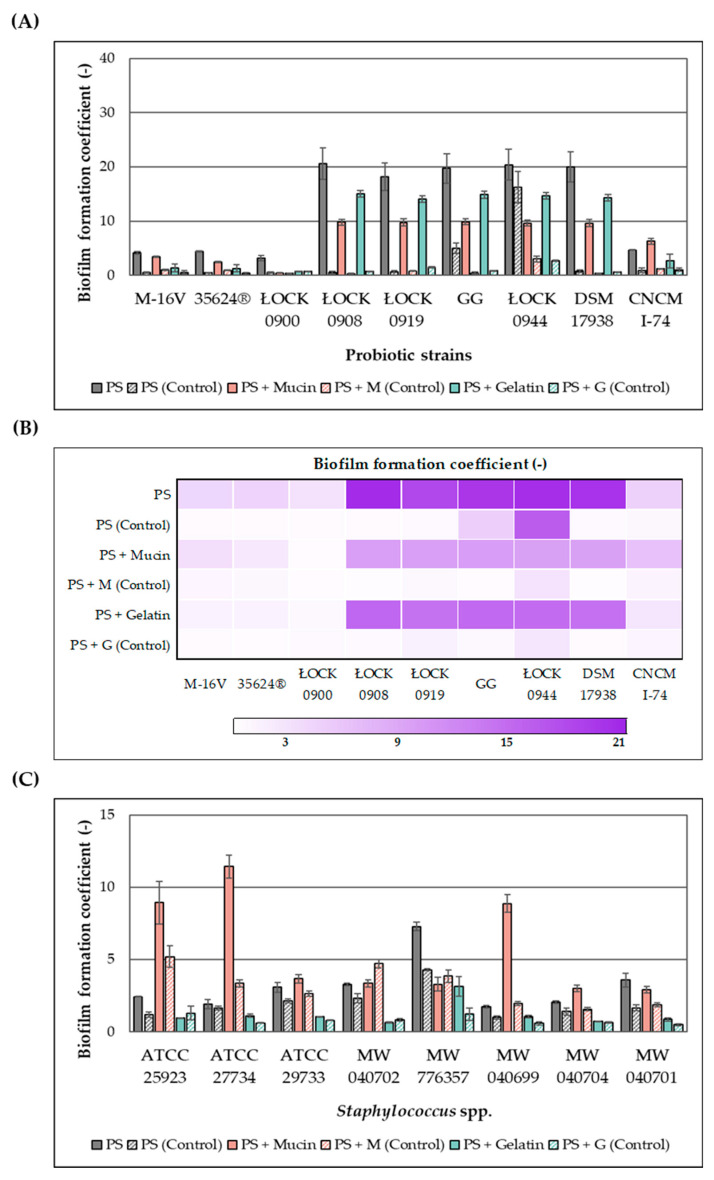

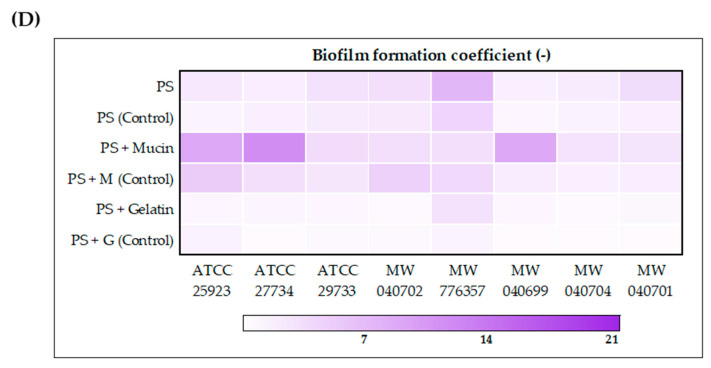
The effect of the ethanolic extract of *H. moscheutos* petals on biofilm formation on abiotic (PS = polystyrene plate) and biotic surfaces (M = mucin, G = gelatin) by: (**A**) probiotic strains—extract conc.: 100.00 mg/mL, except for ŁOCK 0919 (50.00 mg/mL) and ŁOCK 0900 (6.25 mg/mL). (**B**) Heat map illustrating biofilm formation by probiotic strains; (**C**) *Staphylococcus* spp.—extract conc.: 50.00 mg/mL (ATCC 27734 and MW 040699), 25.00 mg/mL (ATCC 25923 and ATCC 29733), 12.50 mg/mL (MW 040702 and MW 040701), 6.25 mg/mL (MW 040704), and 3.13 mg/mL (MW 776357). (**D**) Heat map illustrating biofilm formation by *Staphylococcus* spp. Controls were not treated with the extract.

The effect of the ethanolic extract of *H. moscheutos* petals on biofilm formation by probiotic microorganisms and *Staphylococcus* spp. is presented in Figure 1A–D, respectively, as biofilm formation coefficient (BFC) values. BFC changes were evaluated on abiotic (polystyrene plate (PS)) and biotic (mucin (M) and gelatin (G)) surfaces after 24 h incubation in appropriate media. The results were compared to control samples not treated with the extract. Although the extract generally increased the biofilm formation coefficient, both its stimulatory and inhibitory effects appear to be strain-dependent. Furthermore, the observed response varied depending on the surface type, highlighting the complex nature of this phenomenon and its dependence on multiple factors.

The addition of the extract to the culture medium promoted biofilm formation by probiotic strains. The highest biofilm-forming capacities in the presence of the extract were observed for lactic acid bacteria strains *L. casei* ŁOCK 0908, *L. paracasei* ŁOCK 0919, *L. rhamnosus* GG, *L. brevis* ŁOCK 0944, and *L. reuteri* DSM 17938, with BFC values ranging from 16 to 24 (PS), 9 to 11 (PS + M), and 14 to 16 (PS + G). However, the lowest BFC values were recorded for *B. breve* M-16V, *B. infantis* 35624^®^, and *L. casei* ŁOCK 0900, with values of 3–5 (PS), 0.4–3.5 (PS + M), and 0.7–2 (PS + G), respectively. The probiotic yeast *S. boulardii* CNCM I-745 exhibited intermediate biofilm-forming ability, falling between bifidobacteria and lactic acid bacteria.

In the case of *Staphylococcus* spp., the presence of the extract either enhanced biofilm formation capacity or exerted no statistically significant effect on this phenomenon. Compared to lactic acid bacteria, biofilm formation coefficients measured in the presence of the extract were markedly lower, although in control samples, the pathogens exhibited substantially higher BFC values. Significant differences were also observed in the relationship between BFC and the type of cultivation surface. Generally, the pathogens demonstrated greater biofilm-forming ability on uncoated or mucin-coated surfaces than on gelatin-coated surfaces. On polystyrene plates, the highest biofilm formation in the presence of the extract was observed for *S. capitis* MW 776357 (BFC = 7.3). For *S. aureus* ATCC 29733, *S. aureus* MW 040702, and *S. saprophyticus* MW 040701, BFC values on polystyrene ranged between 3 and 4, while the remaining strains exhibited values close to 2.0. On biotic surfaces, notably high biofilm formation was exhibited by *S. aureus* ATCC 27734, *S. aureus* ATCC 25923, and *S. epidermidis* MW 040699, with BFC values on mucin of 11.4, 8.9, and 8.9, respectively, whereas other strains displayed values ranging from 3 to 4. BFC values on gelatin were 0.5–1.2, except for *S. capitis* MW 776357, which showed a value of 3.1.

### 2.5. Effect of the Extract on Bacterial Autoaggregation

**Figure 2:**
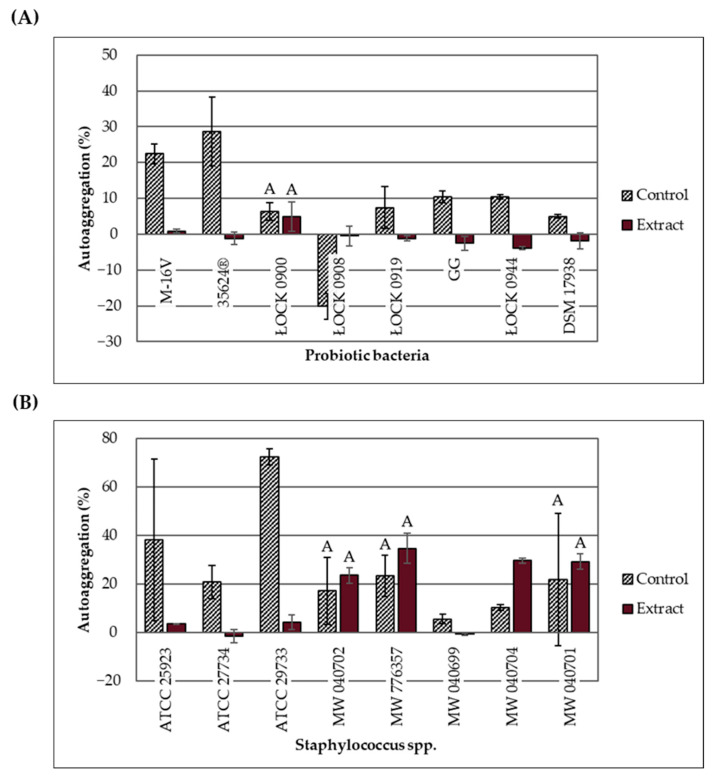
Effect of the *H. moscheutos* petal extract on bacterial autoaggregation ability (4 h, 37 °C): (**A**) probiotic bacteria—extract conc.: 100.00 mg/mL, except for ŁOCK 0919 (50.00 mg/mL) and ŁOCK 0900 (6.25 mg/mL). (**B**) *Staphylococcus* spp.—extract conc.: 50.00 mg/mL (ATCC 27734 and MW 040699), 25.00 mg/mL (ATCC 25923 and ATCC 29733), 12.50 mg/mL (MW 040702 and MW 040701), 6.25 mg/mL (MW 040704), and 3.13 mg/mL (MW 776357). Controls were not treated with the extract. ^A^ No statistically significant differences in the percentage of autoaggregation (% AAg) between extract-treated samples and control (ANOVA, *p* > 0.05; *n* = 3).

Changes in the autoaggregation ability of probiotic bacteria and *Staphylococcus* spp. in response to the ethanolic extract of *H. moscheutos* petals are presented in Figure 2A,B. The percentage of autoaggregation (% AAg) was measured 4 h after mixing the 24 h bacterial cultures. Based on the % AAg values, strains were classified into three groups: high (>70% AAg), moderate (20–70% AAg), and low (<20% AAg) autoaggregation ability [15]. Considering the control samples (without extract), out of the 16 tested strains, one showed high autoaggregation ability (*S. aureus* ATCC 29733), six exhibited moderate ability (both *Bifidobacterium* strains and four pathogenic strains), and nine demonstrated low autoaggregation ability (six lactic acid bacteria strains and three pathogenic strains). The lowest autoaggregation percentage (−20.19%) was recorded for *L. casei* ŁOCK 0908. This strain is known to produce an exopolysaccharide that increases the medium’s viscosity, significantly delaying cell sedimentation.

The effect of the ethanolic extract on bacterial autoaggregation appears to depend not only on strain-specific traits but also on the concentration applied. At concentrations ranging from 25 to 100 mg/mL, the extract significantly reduced % AAg (except for *L. casei* ŁOCK 0908), whereas at concentrations between 3.13 and 12.5 mg/mL, it increased % AAg or had no statistically significant effect. However, the influence of varying extract concentrations on the autoaggregation ability of the same strains remains unclear. The extract significantly increased the autoaggregation percentage of two strains: *S. haemolyticus* MW 040704 (6.25 mg/mL; *p* = 0.003) and *L. casei* ŁOCK 0908 (100 mg/mL; *p* = 0.026). Notably, cultures of strain ŁOCK 0908 supplemented with the extract did not exhibit the high viscosity observed in control samples. It is hypothesized that the extract may affect exopolysaccharide production, reduce medium viscosity, and thus increase % AAg; however, the underlying mechanism remains unknown and warrants further molecular-level investigation.

For *L. casei* ŁOCK 0900 (*p* = 0.745), *S. aureus* MW 040702 (*p* = 0.587), *S. capitis* MW 776357 (*p* = 0.267), and *S. saprophyticus* MW 040701 (*p* = 0.741), the % AAg values did not change significantly. In contrast, the remaining strains exhibited a statistically significant decrease in % AAg (*p* < 0.05). In cultures supplemented with the extract, four pathogenic strains (*S. capitis* MW 776357, *S. haemolyticus* MW 040704, *S. saprophyticus* MW 040701, and *S. aureus* MW 040702) showed moderate autoaggregation, while the remaining 12 strains, including all probiotics, demonstrated low autoaggregation.

### 2.6. Prebiotic Activity

Table 3 presents the prebiotic index (PI) values for all probiotic strains, calculated relative to different *E. coli* strains. The calculations were based on data obtained using a uniform concentration of the extract. PI values were close to zero—negative with respect to the ATCC 11303 strain and positive in relation to ATCC 35218. Low or negative PI values were observed because the tested probiotic strains exhibited poorer growth (based on A_630_ measurements) in the medium supplemented with the prebiotic (extract) compared to the control medium, or their growth was lower than that of the *E. coli* strains in the presence of the extract. Therefore, it was concluded that the ethanol extract of *H. moscheutos* petals did not exhibit prebiotic activity under the tested conditions.

### 2.7. Genotoxicity

Table 4 presents the mean number of revertants per plate, standard deviation, and the mutagenicity index (MI) following treatment with various doses of *H. moscheutos* petals extract, as observed in *Salmonella* Typhimurium strains TA98 and TA100, both with (+S9) and without (−S9) metabolic activation.

The extract induced a reproducible, dose-dependent increase in the number of revertant colonies only in the *S.* Typhimurium TA98 strain. However, revertant counts exceeding twice the control value were observed only at concentrations of 5 and 10 mg/plate. It is important to note that these represent the maximum tested dose levels for non-toxic substances in the Ames test [16]. Therefore, the *H. moscheutos* flower petal extract can be classified as a weak mutagen or non-mutagenic, as supported by the results obtained for the *S.* Typhimurium TA100 strain.

### 2.8. Anti-Inflammatory Properties

The in vitro anti-inflammatory activity of the *H. moscheutos* petals ethanol extract, determined by the egg white albumin denaturation method, is presented in Table 5. At concentrations of 100, 250, and 500 µg/mL, the extract inhibited protein denaturation by 4.73, 31.02, and 54.70%, respectively. For comparison, sodium diclofenac (DCF-Na) showed inhibition levels of 25.15, 33.26, and 56.82% at the same concentrations. No statistically significant differences were observed in the percentage of protein denaturation inhibition between the extract and DCF-Na at 250 and 500 µg/mL. These results indicate that the extract, at these doses, exhibits in vitro anti-inflammatory activity comparable to that of the reference compound with confirmed anti-inflammatory properties.

## 3. Discussion

The most commonly used herbal raw material from the *Hibiscus* genus is the flower, which is widely processed into teas, soft drinks, jellies, dietary supplements, and various other products. *Hibiscus* flowers have long been utilized in traditional medicine for the treatment of skin disorders, biliary tract diseases, cancer, cough, fatigue, gastrointestinal ailments, fever, cardiovascular disorders, neurosis, scurvy, and urinary tract infections. Among the genus, the species most frequently applied in traditional medicine and the food industry include *H. acetosella*, *H. cannabinus* L., *H. diversifolius*, *H. heterophyllus*, *H. moscheutos* L., *H. mutabilis*, *H. rosa-sinensis* L., *H. sabdariffa* L., *H. sinosyriacus*, *H. syriacus* L., *H. tiliaceus* L., and *H. trionum* L. [17]. These species are known to contain a diverse array of bioactive compounds, typically produced via secondary metabolism, including flavonoids, anthocyanins, alkaloids, terpenoids, and phenolic acids, which exert notable physiological effects. Previous studies have quantified the phenolic content in various *Hibiscus* species. Santos Nascimento et al. [18] reported that ethanolic extracts of *H. roseus* flowers contained 11.96 mg/g DW (dry weight) of total polyphenols, 6.26 mg/g DW of flavonoids, and 0.35 mg/g DW of anthocyanins. Similarly, Umaru [19] found that methanolic extracts of *H. moscheutos* contained 48.86 mg/g of total phenolics, 22.23 mg/g of flavonoids, 6.41 mg/g of flavonols, and 1.68 mg/g of anthocyanins. In the present study, significantly higher polyphenol concentrations were observed in the extract. The ethanolic extract of *H. moscheutos* petals contained 216.39–219.52 mg/g FDE (freeze-dried extract) of phenolic compounds, including 93.09 mg/g FDE of flavonols, 59.18 mg/g FDE of flavanols, and 62.08 mg/g FDE of anthocyanins. Chatepa et al. [20] further demonstrated that the type and polarity of the solvent can affect both the composition and phenolic content of extracts. Methanol extracts from two *H. sabdariffa* varieties contained higher concentrations of phenolic compounds and anthocyanins compared to aqueous extracts. Additionally, red calyces consistently exhibited higher total phenolic content than dark red calyces, irrespective of the solvent.

Compared to conventional hibiscus-based food products, such as commercial fruit teas (100% flowers), which contain on average 0.2 mg GAE/mL [21], the analyzed extract of *H. moscheutos* petals exhibited a pronouncedly higher total polyphenol content. This indicates that standard products may not fully reproduce the extract’s bioactive properties. Nonetheless, the extract holds potential for the development of novel functional foods and dietary supplements, although its applications require further investigation.

**Figure 3:**
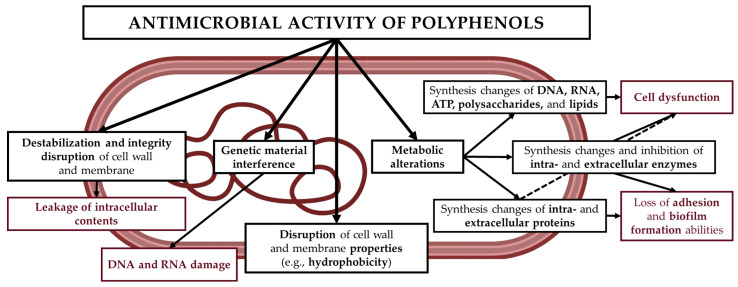
Mechanism of polyphenols’ antimicrobial activity.

One of the key properties of plant extracts is their antagonistic activity against pathogenic and food-contaminating microorganisms. They are effective against microbes that are difficult to eliminate with a single synthetic drug, as their activity results from the synergistic effect of multiple compounds, such as polyphenols, which limits the development of resistance [3,5]. Phenolic compounds exhibit antimicrobial effects through multiple mechanisms, based on their structure and physicochemical interactions with microbial cell components (Figure 3). These include disruption of cell membrane permeability (procyanidins, epigallocatechin gallate, and flavones); interference with genetic material and inhibition of DNA and RNA synthesis (catechin and (-)-epigallocatechin); inhibition of cell wall and lipopolysaccharide (LPS) synthesis (procyanidins); formation of pores in the cell membrane and wall, leading to leakage of intracellular contents (ferulic acid, gallic acid, and p-coumaric acid); inhibition of DNA gyrase (coumarin, quercetin, and catechin); inhibition of efflux pumps (daidzein, genistein, and kaempferol); and inhibition of intracellular enzymes (resveratrol, tannins, and quercetin) [22]. In addition, plant extracts may also affect the probiotic microbiota, exerting either beneficial or adverse effects. Nevertheless, further research is necessary.

The ethanol extract of *H. moscheutos* petals exhibited selective antibacterial activity at a concentration of 100 mg/mL. Many of the tested *Staphylococcus* spp. strains were sensitive to its action, with efficacy appearing to depend on specific strain characteristics. *S. capitis* MW 776357 showed high sensitivity (31.3 ± 3.0 mm), whereas *S. aureus* strains (ATCC 27734, ATCC 29733, and MW 040702), *S. epidermidis* MW 040699, and *S. haemolyticus* MW 040704 exhibited moderate sensitivity (15–24 mm). *S. aureus* ATCC 25923 and *S. saprophyticus* MW 040701 displayed low sensitivity (11–14 mm). The antagonistic activity of extracts from leaves and flowers of various hibiscus species (*H. syriacus*, *H. sabdariffa*, and *H. rosa-sinensis*) against staphylococci, particularly *S. aureus*, has been widely reported [3,23,24,25]. Canli et al. [3] used ethanol extracts from leaves and flowers of Syrian hibiscus and observed results consistent with those reported here for *S. aureus* and *S. epidermidis*. They also demonstrated that flower extracts exerted stronger antimicrobial effects than leaf extracts.

Due to their antimicrobial properties, some plant extracts offer promising options for combating infections caused by microorganisms resistant to conventional antibiotics. In this study, our extract at 100 mg/mL exhibited stronger antagonistic effects against *S. capitis* MW 776357 and *S. haemolyticus* MW 040704 than cefoxitin (30 µg). No significant differences were detected between the extract and cefoxitin for *S. aureus* ATCC 27734, *S. epidermidis* MW 040699, and *S. saprophyticus* MW 040701, suggesting that *H. moscheutos* petal extract could serve as an alternative to certain antibiotics against pathogenic staphylococcal strains. Other tested bacteria, including *Enterococcus faecalis*, *Escherichia coli*, and *Salmonella* spp., were resistant even at the highest extract concentration. While it was initially assumed that the extract acts exclusively against Gram-positive bacteria, evidence from other studies indicates a broader spectrum. For instance, Canli et al. [3] reported that ethanol extracts from *H. syriacus* flowers inhibited not only Gram-negative species, including *E. coli*, *Pseudomonas aeruginosa*, *Pseudomonas fluorescens*, *S.* Enteritidis, *S.* Typhimurium, and *Proteus vulgaris*, but also the Gram-positive bacterium *E. faecalis*. Differences between the results likely stem from variations in extract concentration and methodology: Canli et al. applied 6, 10, and 23 mg per disk using the disk diffusion method, whereas the present study used approximately 10 mg per well with a larger diameter in the agar well diffusion assay. Consequently, strains that showed weak activity in the previous study exhibited negligible growth inhibition in the current experiment.

To further assess the spectrum of the extract’s activity, its effects on yeast and filamentous fungi were evaluated. At a concentration of 100 mg/mL, the extract did not demonstrate any antifungal activity against the tested strains. Canli et al. [3] reported moderate susceptibility of the yeast *Candida albicans* DSMZ 1386 to an extract from *H. syriacus* flowers. In contrast, *Candida topicalis* was resistant, as was *Candida vini* ŁOCK 0009 in the present study. These results indicate that susceptibility to the extract within the genus *Candida* might be species- or strain-specific.

Various studies suggest that phenolic plant extracts can have a prebiotic-like effect, maintaining the normal composition of the intestinal microbiota by supporting the growth of beneficial microorganisms, such as lactobacilli and bifidobacteria [26]. The extract of *H. moscheutos* petals did not show antagonistic activity against probiotic *Bifidobacterium* spp. or most lactic acid bacteria strains examined. Among these, only the growth of two of the four *Lacticaseibacillus* strains was inhibited: weakly *L. paracasei* ŁOCK 0919 (13.0 ± 1.4 mm) and moderately *L. casei* ŁOCK 0900 (21.0 ± 1.4 mm). It also did not selectively promote their growth over non-probiotic gut bacteria. Thus, the extract lacked prebiotic activity in vitro. It is worth noting, however, that the assay employed a modified method originally developed for assessing oligosaccharide prebiotic effects, adapted here to evaluate the influence of phenolic compounds and other phytochemicals in the extract. Prebiotic activity in vivo cannot be ruled out, as certain mechanisms may not have been captured by this in vitro approach. A study by Diez-Echave et al. [27] demonstrated that *H. sabdariffa* flower extract (HSE) could modulate the intestinal microbiota in a model of diet-induced obesity. HSE reduced the ratio of *Firmicutes* to *Bacteroidetes* and altered the abundance of various bacterial genera and orders, thereby counteracting obesity-related intestinal dysbiosis, which is considered a pathogenetic factor.

The ability to autoaggregate and form biofilms is one of the most desirable characteristics of probiotic strains, primarily related to their adhesive properties [28]. However, in the case of food-contaminating and pathogenic microbiota, this phenomenon is undesirable. The ability to adhere to biotic surfaces, such as the host’s mucous membranes, and to abiotic surfaces, such as polystyrene, facilitates environmental colonization and promotes the maintenance of a stable microflora. Autoaggregation, resulting from intercellular interactions, may be the first step in biofilm formation—a multicellular structure that often exhibits antagonistic properties toward pathogens. Van der Waals forces (attractive) and electrostatic interactions (repulsive) also affect this process, with their balance determining the strength of bacterial adhesion to surfaces. These properties allow probiotic strains to effectively compete with pathogenic microorganisms for space and nutrients [28,29]. The *H. moscheutos* petal extract modulates bacterial autoaggregation (AAg) in a manner dependent not only on strain characteristics but also on its concentration in the medium. When applied at 25–100 mg/mL, it significantly reduced the percentage of autoaggregation (% AAg), whereas at 3.13–12.5 mg/mL, it either increased % AAg or had no significant effect.

The ethanolic extract of *H. moscheutos* petals increased the biofilm-forming capacity of both probiotic and pathogenic microbiota on abiotic (polystyrene) and biotic (pork mucin and gelatin) surfaces. Compared to lactic acid bacteria, the biofilm formation coefficients (BFC) of *Staphylococcus* species in the presence of the extract were significantly lower, even though these bacteria exhibited higher BFC values in the control samples. Significant differences were also observed depending on the type of culture surface. Staphylococci generally formed more robust biofilms on uncoated or mucin-coated surfaces than on gelatin-coated surfaces.

Biofilms play a crucial role in the pathogenicity of many bacteria, including *Staphylococcus* spp. Previous studies indicate that approximately 43–88% of clinical *S. aureus* isolates are capable of forming biofilms, although this trait exhibits genotypic variability among strains [30]. Biofilm-associated staphylococci display markedly reduced susceptibility to antibiotics compared with their planktonic counterparts. Consequently, the stimulation of biofilm formation observed in pathogenic strains in response to the extract is undesirable, as it can promote the development of chronic and difficult-to-treat infections, necessitating prolonged antimicrobial therapy and, in some cases, surgical intervention [30,31]. These findings raise concerns regarding the safety profile of the extract and might restrict its potential applications in pharmaceutical or medical contexts. Further investigation of these phenomena should be pursued in future research.

Takada et al. [32] investigated the ability of *H. sabdariffa* flower extract to inhibit biofilm formation by oral pathogenic bacteria on polystyrene plates. The extract significantly reduced biofilms formed by *Streptococcus mutans* UA159, *Streptococcus sobrinus* ATCC 33478, *Aggregatibacter actinomycetemcomitans* Y4, *Porphyromonas gingivalis* ATCC 33277, and *Prevotella intermedia* ATCC 25611. For all tested strains, a pronounced inhibitory effect on biofilm formation on abiotic surfaces was observed. Based on these results, the authors suggested that regular consumption of hibiscus extract as a beverage could reduce the risk of oral diseases such as dental caries and periodontal disease. In another study, Majidinia et al. [33] demonstrated that cranberry-derived proanthocyanidins suppressed the swarming motility of *Pseudomonas aeruginosa* and modulated its biofilm formation. At a concentration of 1 mg/mL, the inhibition rate reached 40.9%, while at 10 mg/mL, it increased to 55.7%, accompanied by a reduction in biofilm thickness from 26 μm to 20 μm. This decrease in biofilm density was attributed to differential expression of 159 proteins.

The mechanism by which the extract influences autoaggregation and biofilm formation capacity remains unclear and warrants further molecular investigation. It has been hypothesized that the bioactive components of the extract may alter the chemical composition of the bacterial cell wall, thereby affecting surface physicochemical properties such as hydrophobicity. Probiotics with higher hydrophobicity and greater self-aggregation potential are generally considered to exhibit enhanced adhesion [34]. However, no such relationship was observed in the present study. It should also be emphasized that the analyses were performed only at concentrations corresponding to the highest level at which no growth inhibition of a given microorganism was detected. Consequently, the potential effects of other extract concentrations on the same strains remain unknown.

Genotoxicity is defined as the potentially harmful effect on genetic material resulting from the induction of permanent, heritable changes in the quantity or structure of nucleic acids [35]. This represents a distinct and important type of toxicity, as specific genotoxic events are recognized as hallmarks of cancer. The consequences of DNA damage may include the development or predisposition to disease, increased morbidity and mortality, alterations in hereditary traits, and impaired reproductive capacity [36,37]. Genotoxicity assays are designed to provide information on various types of mutations, including gene mutations, structural chromosomal aberrations, and numerical chromosomal abnormalities [36].

The Ames test exploits the ability of auxotrophic, histidine-dependent mutants of *Salmonella* Typhimurium to revert mutations under the influence of mutagenic agents. *H. moscheutos* petal extract at concentrations ranging from 1 to 10 mg/plate exhibited low genotoxicity in the Ames test. A reproducible, dose-dependent increase in the number of revertant colonies was observed only in the *S.* Typhimurium TA98 strain, with values exceeding twice the control level only at 5 and 10 mg/plate. Similar results were reported by Chewonarin et al. [38] for the *S.* Typhimurium TA98 strain using ethanolic extracts of *H. sabdariffa* flowers at concentrations of 3.125–50 mg/plate. At the highest concentration (50 mg/plate), complete inhibition of bacterial growth was observed for *S.* Typhimurium TA98 and TA100. However, no genotoxic effect was detected for *S.* Typhimurium TA100 up to 25 mg/plate without metabolic activation (−S9).

Our study likewise demonstrated no mutagenic activity of the extract against *S.* Typhimurium TA100, both with metabolic activation (+S9) and without activation (−S9), within the concentration range of 1–10 mg/plate. It is important to emphasize that these values correspond to the maximum dose levels recommended in the test protocol for non-toxic compounds. For comparison, Rosa et al. [39] reported that methanolic extracts of *H. tiliaceus* flowers did not exhibit mutagenic activity in the Ames test.

Genotoxicity is a critical aspect of the safety assessment of plant extracts. To ensure their safe use, it is essential to identify potential sources of genotoxic activity, evaluate the influence of processing methods, and guarantee appropriate data interpretation by regulatory authorities. Preliminary findings indicating low genotoxicity of ethanolic extracts of *Hibiscus* spp., even at relatively high concentrations, are encouraging. Nevertheless, further comprehensive investigations, including long-term studies and assessments in diverse biological models, are required to confirm their safety profile before application in the development of new therapeutic agents or functional foods.

The ethanolic extract of *H. moscheutos* petals showed good anti-inflammatory properties in an in vitro model. At concentrations of 250 and 500 μg/mL, its effect was comparable to that of the nonsteroidal anti-inflammatory drug diclofenac sodium at the same levels. Protein denaturation inhibition reached 4.73%, 31.02%, and 54.70% at 100, 250, and 500 μg/mL, respectively. These values were similar to those obtained for diclofenac sodium, consistent with the results reported in the paper from which the method was adapted [40]. Furthermore, Singh et al. [41] reported that hibiscus infusion (56%) showed lower inhibition of protein denaturation compared to green tea (91%), chamomile tea (88%), ginger tea (91%), and rose tea (90%). Sruthi et al. [42] also found comparable in vitro anti-inflammatory activity between diclofenac and the ethanolic extract of *H. rosa-sinensis* leaves.

Mechanistic studies have demonstrated that anthocyanins are promising natural agents against inflammation [43]. The effects of various fruit- and vegetable-derived extracts rich in anthocyanin fractions (ranging from 4.9 to 38.5 mg/g dry weight) were investigated in human endothelial cells by assessing their impact on the expression of endothelial adhesion molecules VCAM-1 and ICAM-1. All anthocyanin-rich extracts exhibited biological activity, with antioxidant capacity varying in strength. Extracts containing non-acylated anthocyanins (typical of fruits) showed stronger effects compared to those with more complex aromatic acylated anthocyanins (common in vegetables), particularly those acylated with cinnamic acid. Accordingly, extracts with non-acylated anthocyanins more effectively reduced the expression of pro-inflammatory endothelial markers than their acylated counterparts, suggesting a structure-dependent protective role of anthocyanins in inflammation. On the other hand, the study by Zhong et al. [44] focused on three structurally related flavonols—fisetin, quercetin, and myricetin—in LPS-stimulated RAW264.7 murine macrophages. These flavonols share a common backbone but differ in the number and position of hydroxyl groups. Their capacity to modulate inflammatory responses was influenced by these structural differences. All compounds inhibited excessive nitric oxide (NO) production, with fisetin showing the highest activity, reaching 52% at a concentration of 20 μM. In addition, the flavonols reduced intracellular levels of ROS, TNF-α, and IL-6. Mechanistically, they suppressed the activation of the NF-κB and MAPK signaling pathways by inhibiting the phosphorylation of IκBα, p65, JNK, ERK, p38, and MEK and by reducing the nuclear translocation of NF-κB p65.

In our in vitro study, the tested extract showed anti-inflammatory activity comparable to diclofenac sodium, supporting further mechanistic studies aimed at elucidating the underlying molecular pathways and potential in vivo effects.

Plant extracts from the *Hibiscus* genus flowers, as a concentrated source of valuable compounds, may be a promising source of new drugs, supplements, and food additives. This thesis is supported by published studies indicating their effectiveness in treating many modern diseases [4,5,45].

## 4. Materials and Methods

### 4.1. Plant Material

Flower petals of *H. moscheutos* L., free from visible mechanical damage, signs of pathogen infection, or insect herbivory, were collected between July and September 2024 from a privately maintained cultivation in Łódź, Poland. The plants were grown in pots under southern sun exposure and had deep burgundy flowers.

### 4.2. Preparation of Hibiscus Petal Extract

Air-dried flower petals were ground using a G21 Perfect Smoothie Vitality blender (G21-Vitality, Katovice, Czech Republic). Approximately 36 g of the material was then placed in a sealed polyethylene container and extracted at a ratio of 1:10 (*w*/*v*) using a mixture of ethanol, water, and formic acid in a volumetric ratio of 50:49.8:0.2. Extraction was carried out at room temperature for 1 h in an ULTRON ultrasonic bath (ULTRON Electronic Devices Plant, Dywity, Poland), after which the solution was separated from the solid fraction by filtration. The remaining solid fraction was subjected to two additional extractions following the same protocol. The combined extracts were concentrated using a Heidolph Hei-VAP rotary evaporator (Heidolph Instruments GmbH & Co.KG, Schwabach, Germany) and then freeze-dried using a CHRIST Alpha 1-2 LDplus lyophilizer (Sigma Laborzentrifugen GmbH, Osterode am Harz, Germany). The dried extract was stored in a tightly sealed container placed inside a polyethylene bag with a desiccant at −20 °C.

### 4.3. Qualitative and Quantitative Analysis of Phenolic Compounds HPLC-DAD-MS (Anthocyanins, Phenolic Acids, and Flavonols)

Approximately 5 mg of the extract was weighed using an analytical balance and dissolved in 5 mL of methanol:water:formic acid (70:29.8:0.2, *v*/*v*/*v*). Prior to chromatographic analysis, the samples were diluted 1:1 (*v*/*v*) with mobile phase A and centrifuged at 12,000× *g*.

Analysis of anthocyanins, phenolic acids, and flavonols in the extract was performed using the LC-DAD-MS technique as described by Sójka et al. [46]. A Dionex Ultimate 3000 UHPLC system equipped with a diode array detector (DAD) and a Q Exactive Orbitrap mass spectrometer (Thermo Fisher Scientific, Waltham, MA, USA) was used. Mobile phase A consisted of 1% formic acid in water, and mobile phase B was 1% formic acid in methanol. The separation gradient was as follows: 0–30 min, 20–65% (*v*/*v*) B; 30–31 min, 65–100% (*v*/*v*) B; 31–33 min, 100% (*v*/*v*) B; 33–34 min, 100–20% (*v*/*v*) B; and 34–45 min, 20% (*v*/*v*) B. The test compounds were separated on a Gemini-NX C18 column (150 mm × 4.6 mm i.d., 3 µm particle size) with a 4 mm × 3 mm i.d. precolumn (Phenomenex, Torrance, CA, USA). The column temperature was maintained at 35 °C, the flow rate was set to 0.5 mL/min, and the injection volume was 10 µL. The following mass detector parameters were used: positive and negative ionization modes, vaporizer temperature of 400 °C, ion spray voltage of 3.8 kV, capillary temperature of 380 °C, and sheath gas and auxiliary gas flow rates of 60 and 20 units, respectively. The detector was operated in either full MS or full MS/dd-MS2 mode. The full MS mode employed a scan range of *m*/*z* 100–1000. To generate MS2 data, the NCE (Normalized Collision Energy) parameter was set to 20 eV. Identification was performed based on standards: cyanidin-3-glucoside, delphinidin-3-glucoside (Extrasynthese, Genay, France), chlorogenic acid, and quercetin-3-rutinoside (Sigma-Aldrich, Steinheim, Germany), as well as MS data and available literature. Quantitative analysis was carried out using calibration curves constructed with the above-mentioned standards, detecting at 520 nm for anthocyanins, 360 nm for flavonols, and 320 nm for hydroxycinnamic acids. All samples were analyzed in four replicates.

### 4.4. HPLC-FD Analysis of Flavanols (Sums of Proanthocyanidins and Catechins)

Quantification of flavan-3-ols was carried out by acid-catalysed degradation of polymeric proanthocyanidins in an excess of phloroglucinol. This determination was performed using a modified method described by Kennedy and Jones [47]. A detailed description of the modification was described in our previous publications [48,49]. For the phloroglucinolysis reaction, 10 mg of lyophilised extract was used. Free catechins were determined in a solution obtained by dissolving 5 g of lyophilized extract in 2 mL of a methanol:water:formic acid mixture in a ratio of 70:29.9:0.1 For the analysis of phloroglucinolysis reaction products and free catechins, the same chromatographic conditions (column, gradient, mobile phases, and standards) were applied using a Shimadzu HPLC system (Tokyo, Japan), consisting of an LC-20AD pump, DGU-20ASR degasser, CTO-10AS column oven, SIL-20AC autosampler, and an RF-10AXL fluorescence detector, as described by Karlińska et al. [43]. All samples were analysed in four replicates.

### 4.5. Spectrophotometric Determination of Phenolic Compounds (Folin–Ciocalteu Method)

To determine the total polyphenol content using the Folin–Ciocalteu method, 5 mg of lyophilised extract was dissolved in 2 mL of a methanol:water:formic acid mixture (70:29.9:0.1, *v*/*v*/*v*). Total phenolics were measured according to the method described by Singleton and Rossi [50], with slight modifications as previously reported in our publication [51]. Results were expressed as mg of gallic acid equivalents (GAE) per gram of lyophilised extract. All samples were analyzed in quadruplicate.

### 4.6. Test Organisms

To assess the antagonistic and prebiotic activity of the ethanolic *Hibiscus* petal extract, a total of 38 microbial strains were used, including 28 bacterial strains (23 Gram-positive and 5 Gram-negative) and 10 fungal strains. Nine of the tested strains had documented probiotic properties. These included six strains of lactic acid bacteria: *Lacticaseibacillus casei* (ŁOCK 0900 and ŁOCK 0908), *Lacticaseibacillus paracasei* (ŁOCK 0919), *Lacticaseibacillus rhamnosus* GG (ATCC 53103), *Levilactobacillus brevis* (ŁOCK 0944), and *Limosilactobacillus reuteri* (DSM 17938); two strains of *Bifidobacterium*: *B. breve* M-16V and *B. longum* subsp. *infantis* 35624 (hereinafter referred to as *B. infantis*); and one strain of yeast: *Saccharomyces cerevisiae* var. *boulardii* (CNCM I-745, hereinafter referred to as *S. boulardii*).

The remaining bacterial group consisted of pathogenic and food-contaminating strains, including Gram-negative strains—*Escherichia coli* (ATCC 11303 and ATCC 35218), *Salmonella enterica* subsp. *enterica* serovar Choleraesuis (ATCC 7001), serovar Enteritidis (ATCC 13076), and serovar Typhimurium (ATCC 14028), as well as Gram-positive strains—*Enterococcus faecalis* (ATCC 29212 and ATCC 51299), *Staphylococcus aureus* (ATCC 25923, ATCC 27734, ATCC 29733, and MW 040702—NCBI accession number), *Staphylococcus capitis* (MW 776357), *Staphylococcus epidermidis* (MW 040699, MW 040700, and MW 040703), *Staphylococcus haemolyticus* (MW 040704 and MW 776358), *Staphylococcus saprophyticus* (MW 040701), *Staphylococcus warneri* (MW 776360), and *Staphylococcus xylosus* (MW 776359).

The fungi selected for this study were either used in food production or associated with food spoilage and contamination, with the exception of *S. boulardii*, which is probiotic. In total, three yeast strains were tested: *Candida vini* (ŁOCK 0009), *Saccharomyces cerevisiae*, and *Saccharomyces boulardii* (CNCM I-745), as well as seven filamentous fungal strains: *Aspergillus niger* (ŁOCK 0431), *Aspergillus oryzae* (ŁOCK 0445), *Geotrichum candidum* (ŁOCK 0511), *Mucor hiemalis* (ŁOCK 0519), *Penicillium candidum*, *Penicillium chrysogenum* (ŁOCK 0532), and *Rhizopus oryzae* (ŁOCK 0550), in the context of assessing the antifungal properties of the extract.

Many of the strains originated from the ŁOCK 105 Collection of Pure Cultures of Industrial Microorganisms, belonging to the Institute of Fermentation Technology and Microbiology (Lodz University of Technology, Lodz, Poland), or were purchased from the American Type Culture Collection (ATCC, Manassas, VA, USA). The remaining microorganisms were isolated from commercial starter cultures, dietary supplements, probiotic preparations, as well as from food and human skin (Table 6). Identification of environmental isolates was based on phenotypic characteristics. Their species affiliation was confirmed using molecular methods based on 16S ribosomal RNA gene sequence analysis [52,53].

Bacteria were stored in cryobanks at −20 °C. Before testing, they were activated by subculturing into the following media: Lactic acid bacteria were cultured in MRS broth (Merck, Darmstadt, Germany); bifidobacteria were cultured in a liquid medium prepared according to a modified composition of BSC Propionate Agar Base (HiMedia, Mumbai, India), with raffinose replacing galactooligosaccharide and without agar supplementation (hereinafter referred to as modified BSC broth); and pathogenic and foodborne contaminant bacteria were cultured on slants of Nutrient Agar (Merck, Darmstadt, Germany).

Yeasts and molds were stored at 4 °C on Sabouraud-2% Dextrose Broth (Merck, Darmstadt, Germany) solidified with 2% agar (hereinafter referred to as SDA) and activated by subculturing onto a fresh SDA plate.

Directly before each assay, fresh microbial suspensions (inocula) were prepared by resuspending centrifuged cell pellets (7000 rpm, 5 min) or material collected with a loop from solid media in 0.9% (*w*/*v*) saline solution. The turbidity of the suspensions was standardized to 1.0 on the McFarland scale using a DEN-1B densitometer (Biosan, Riga, Lithuania) or to 1–3 × 10^8^ cells/mL using a Thoma counting chamber (for fungi). Biomass was collected from 24–72 h cultures incubated at 37 °C (bacteria and *S. boulardii*) or 30 °C (other fungi). For *Bifidobacterium* species, cultures were additionally incubated in an anaerostat (<0.1% O_2_; 7–15% *v*/*v* CO_2_) at the same temperature.

### 4.7. Antimicrobial Activity

The antagonistic activity of the ethanol extract from *H. moscheutos* petals was evaluated against the test organisms described above using the agar well diffusion method, following the protocols of Begashaw et al. [54] and Uddin et al. [55] with minor modifications. Each agar plate was prepared by pouring approximately 25 mL of MRS agar (Merck, Darmstadt, Germany) for probiotic bacteria, Mueller–Hinton agar (BTL, Lodz, Poland; hereinafter MHA) for pathogenic bacteria, or SDA for fungi into 10 cm diameter Petri dishes, resulting in an agar layer approximately 3–4 mm thick. Microbial suspensions were evenly spread over the solidified and slightly dried medium surface by streaking in three directions to form a uniform lawn. Wells of 10 mm diameter were then punched into the agar using a cork borer, and 100 μL of extract solution at 100 mg/mL (prepared in 5% (*v*/*v*) DMSO) was added to each well. Positive controls consisted of antibiotic discs (6 mm diameter) impregnated with amoxicillin (10 μg), ampicillin (2 μg), cefoxitin (30 μg), chloramphenicol (30 μg), ciprofloxacin (5 μg), gentamicin (120 μg), or penicillin (10 μg), while nystatin (0.21% *w*/*v*) was also added to wells. A 5% (*v*/*v*) DMSO solution served as the negative control. Plates were incubated for 18–24 h at 37 °C (for bacteria and *S. boulardii*) or 30 °C (for other fungi), with *Bifidobacterium* cultures maintained anaerobically (<0.1% *v*/*v* O_2_; 7–15% *v*/*v* CO_2_).

The diameter of the inhibition zones was measured with an accuracy of 1 mm [54,55]. The presence of a distinct inhibition zone with a diameter ≥ 11 mm around the wells was considered indicative of significant microbial sensitivity to the extract at the tested concentration [54,56]. The results obtained were compared to those of the antibiotic discs. Tests were conducted in at least two replicates, and the mean value (in mm) ± standard deviation was used for analysis. All experiments were carried out under strict aseptic conditions.

### 4.8. Minimum Inhibitory Concentrations (MICs)

Minimum inhibitory concentration (MIC) was defined as the lowest concentration of an antimicrobial agent (extract) that significantly inhibited microbial growth, indicated by a clear inhibition zone with a diameter ≥ 11 mm after 24 h of incubation [54,56,57]. MIC was determined using the previously described agar well diffusion method, with minor modifications based on Gonelimali et al. [58]. Only strains against which the extract showed antagonistic activity at 100 mg/mL were tested. For other microorganisms, MIC was assumed to be >100 mg/mL. Into wells (⌀ 10 mm) punched in the agar medium, 100 μL of extract solutions at concentrations of 100, 50, 25, 12.5, 6.25, or 3.125 mg/mL, prepared by twofold serial dilutions in 5% (*v*/*v*) DMSO solution, were added. A 5% (*v*/*v*) DMSO solution was used as the negative control. Plates were incubated for 18–24 h at 37 °C.

### 4.9. Biofilm Formation

The effect of the extract from *H. moscheutos* petals on biofilm formation by probiotic microbiota and *Staphylococcus* spp. (sensitive to extract concentrations ≤ 100 mg/mL) on abiotic and biotic surfaces was investigated using a modified method described by Stepanović et al. [59]. Abiotic surfaces consisted of sterile polystyrene plates (24-well, tissue culture plates; Jet Biofil, Guangzhou, China) with flat bottoms, while biotic surfaces were the same plates coated with a layer of mucin or gelatin.

Plates (24-well, tissue culture plates; Riomavix, Madrit, Spain) were coated by adding 200 μL of porcine mucin (Sigma-Aldrich, St. Louis, MO, USA) or pharmaceutical gelatin (Sigma-Aldrich, St. Louis, MO, USA) (1 mg/mL) into each well, followed by shaking at 100 rpm for 6 h at room temperature. Then, plates were incubated for 18 h at 4 °C. After incubation, each well was washed three times with 200 μL of sterile PBS buffer (0.1 M; pH 7.0).

Into each well, both coated and uncoated, 200 μL of growth medium was added: MRS broth for lactic acid bacteria, modified BSC broth for *Bifidobacterium* spp., YPG for *S. boulardii*, or Nutrient broth for *Staphylococcus* spp., along with 10 μL of microbial suspension and 100 μL of extract solution at the highest concentration at which no growth inhibition was observed, or 100 μL of sterilized (121 °C, 15 min) distilled water. For negative controls, 10 μL of inoculum was replaced by 10 μL of sterile distilled water. This experiment was performed in triplicate. Plates were incubated for 24 h at 37 °C, with *Bifidobacterium* cultures incubated anaerobically (<0.1% *v*/*v* O_2_; 7–15% *v*/*v* CO_2_) at the same temperature.

After incubation, the growth medium was removed, and each well was washed three times with 200 μL of PBS buffer (0.1 M; pH 7.0) to remove non-adherent cells. Then, each well was stained with 300 μL of 0.1% (*w*/*v*) aqueous crystal violet solution. Plates were shaken at 150 rpm for 45 min at room temperature, then washed again with PBS buffer (0.1 M; pH 7.0). Crystal violet was extracted from the wells using 200 μL of 99% ethanol, and absorbance was measured at 492 nm using a 96-well plate reader (Rayto RT-6900 Microplate Reader, Shenzhen, China), with 99% (*v*/*v*) ethanol as the blank.

The biofilm formation coefficient (BFC) was calculated according to the formula proposed by Piekarska-Radzik and Klewicka [28]:

(1) $$BFC=\frac{A492}{{A492}_{NC}}$$

where A492—absorbance of the test sample (with or without the addition of extract) and A492_NC_—absorbance of the negative control with uninoculated medium.

### 4.10. Autoaggregation

The effect of *H. moscheutos* petals extract on the autoaggregation of probiotic strains and selected pathogens sensitive to extract concentrations ≤ 100 mg/mL was determined using a modified method described by Rahman et al. [15]. After 24 h of incubation of the medium inoculated with the microbial suspension (with or without the extract) in polypropylene tubes (95 × 17 mm), the culture was vortexed for 15 s and then left to stand for 4 h at 37 °C. Bifidobacteria were incubated anaerobically in an anaerostat (<0.1% O_2_; 7–15% *v*/*v* CO_2_). The media—MRS broth (for lactic acid bacteria), modified BSC broth (for *Bifidobacterium* spp.), YPG (for *S. boulardii*), and Nutrient broth (for pathogens)—were supplemented with the extract at the highest concentration at which no growth inhibition of the given microorganism was observed. Negative controls consisted of uninoculated media. All assays were performed in triplicate. Absorbance was measured at a wavelength of 630 nm after 0 and 4 h of incubation using a Rayto RT-6900 Microplate Reader (Shenzhen, China), by taking 200 μL samples from 3 to 4 mm below the liquid surface into a 96-well plate.

The autoaggregation ability of the strains was expressed as the percentage of autoaggregation (% AAg) and calculated using the following formula:

(2) $$\%AAg=\left(\frac{{A630}_{0}-{A630}_{4}}{{A630}_{0}}\right)\cdot 100$$

where A630_0_ and A630_4_ represent the absorbance of the culture at the beginning of incubation (0 h) and after 4 h, respectively. Based on the % AAg values, the autoaggregation ability of the strains, both in the absence and presence of the extract, was classified according to the criteria adopted by Rahman et al. [15] into three groups: high (>70% AAg), medium (20–70% AAg), and low (<20% AAg).

### 4.11. Prebiotic Activity

Prebiotic activity (prebiotic index) is defined as the ability of a given substrate to selectively stimulate the growth of probiotic microorganisms compared to other non-probiotic gut microbes and in the presence of a non-prebiotic substrate such as glucose [60]. Cell suspensions (10 μL) were added to wells of a 96-well microplate containing 190 μL of the appropriate growth medium: MRS broth (for lactic acid bacteria), modified BSC broth (for *Bifidobacterium* spp.), YPG (for *S. boulardii*), or Nutrient broth supplemented with 2% glucose (for *E. coli*). The media were supplemented with the extract at the highest concentration at which no inhibition of probiotic growth was observed. Control samples contained media without the extract. The samples were incubated at 37 °C in an incubator, except for bifidobacteria, which were cultured anaerobically (<0.1% *v*/*v* O_2_; 7–15% *v*/*v* CO_2_) at the same temperature. All tests were conducted in triplicate. For each strain, absorbance was measured at a wavelength of 630 nm at 0 and 24 h post-inoculation using a Rayto RT-6900 Microplate Reader (Shenzhen, China).

The prebiotic index (PI) for all probiotics in combination with different *E. coli* strains was calculated according to the method described by Huebner et al. [60]:

(3) $$PI=\left(\frac{∆Probiotic\;Extract\;A630}{∆Probiotic\;Control\;A630}\right)-\left(\frac{∆E.\;coli\;Extract\;A630}{∆E.\;coli\;Control\;A630}\right)$$

where Δ Probiotic Extract A630—difference in absorbance between 0 and 24 h of probiotic strain culture in the presence of the extract, Δ Probiotic Control A630—difference in absorbance between 0 and 24 h of probiotic strain culture without the extract, Δ *E. coli* Extract A630—difference in absorbance between 0 and 24 h of *E. coli* strain culture in the presence of the extract, and Δ *E. coli* Control A630—difference in absorbance between 0 and 24 h of *E. coli* strain culture without the extract.

### 4.12. Genotoxicity

The mutagenic (genotoxic) activity of the *H. moscheutos* petals extract was determined using the Ames test with histidine-dependent *Salmonella* Typhimurium mutants TA98 and TA100, both with (+S9) and without (–S9) metabolic activation, following the method described by Maron and Ames [61]. The test strains were purchased as lyophilized preparations on Lyophilized STDiscs (MOLTOX^®^ Molecular Toxicology, Boone, NC, USA) and stored at 4 °C. Overnight cultures were prepared in Nutrient broth No. 2 medium (Thermo Scientific, Waltham, MA, USA), incubated at 37 °C for 12–16 h, followed by an additional 2 h of shaking at 100 rpm, until a density of 1 × 10^9^ cells/mL was reached. The metabolic activation of promutagenic compounds into mutagens was conducted in the presence of oxidative enzymes contained in the mammalian liver extract (the S9 microsomal fraction—Mutazyme™ 10% S-9 Mix (MOLTOX^®^ Molecular Toxicology, Boone, NC, USA)). A fresh S9 mix was prepared before each test according to the manufacturer’s instructions.

Mutagenicity of the extract was tested at doses of 1, 5, and 10 mg per plate. Solutions of varying concentrations of the extract, prepared in 5% (*v*/*v*) DMSO (Sigma-Aldrich, Saint-Quentin-Fallavier, France), were added to 0.5 mL of 0.2 M PBS buffer (Th. Gayer GmbH & Co. KG, Renningen, Germany) or to 0.5 mL of the S9 mix, together with 0.1 mL of bacterial culture, and incubated at 37 °C for 15 min. After incubation, 2 mL of Top Agar (sodium chloride (Chempur, Piekary Śląskie, Poland): 5 g/L; agar (Bio-Maxima, Lublin, Poland): 6 g/L), supplemented with histidine (Sigma-Aldrich, St. Louis, MO, USA) (0.096 mg/100 mL) and biotin (Sigma-Aldrich, St. Louis, MO, USA) (0.1236 mg/100 mL) at 45 °C, was added. The mixture was thoroughly mixed and evenly spread over the surface of Minimal Glucose Agar (glucose (Chempur, Piekary Śląskie, Poland) 40%: 50 mL/L; Vogel-Bonner E50X medium: 20 mL/L; agar: 15 g/L).

Diagnostic mutagens—daunomycin (MOLTOX^®^ Molecular Toxicology, Boone, NC, USA) (6 μg/plate) for strain TA98 and sodium azide (MOLTOX^®^ Molecular Toxicology, Boone, NC, USA) (1.5 μg/plate) for strain TA100—were used as positive controls. An experiment performed identically, but with sterile distilled water instead of the extract, served as a negative control. All assays were performed in triplicate. Plates were incubated at 37 °C for 48 h, after which the number of colonies exhibiting His^+^ reversion mutations was counted and compared to the negative control, which showed spontaneous His^+^ reversion. Results were expressed as the mean number of colony-forming units per plate (CFU/plate) ± standard deviation.

### 4.13. Anti-Inflammatory Properties

The anti-inflammatory properties of *H. moscheutos* petals extract were determined in vitro using the egg white albumin denaturation method described by Rahman et al. [40]. Reaction mixtures of 5 mL were prepared from 0.2 mL of egg white (obtained from a fresh egg), 2.8 mL of PBS buffer (0.1 M; pH 6.4), and 2 mL of the test extract solution at various concentrations (100, 250, and 500 μg/mL in 5% (*v*/*v*) DMSO solution). An analogous volume of distilled water was used as a control. The samples were then incubated in a water bath at (37 ± 2) °C for 15 min, followed by heating at 70 °C for 5 min. After cooling, the samples were mixed thoroughly, and their absorbance was measured at a wavelength of λ = 660 nm using a Rayleigh UV-2601 spectrophotometer (Rayleigh, Beijing, China), using an empty cuvette as a blank. Diclofenac sodium (DCF-Na) (Sigma-Aldrich, St. Louis, MO, USA), at the same concentrations (100, 250, and 500 μg/mL), was used as an anti-inflammatory reference compound and subjected to the same procedures for absorbance determination. This experiment was performed in triplicate.

### 4.14. Statistical Analysis

The obtained results were analyzed using one-way analysis of variance (one-way ANOVA), followed by Tukey’s post-hoc test for pairwise comparisons of means. Values of *p* < 0.05 were considered statistically significant. All data are presented as mean ± standard deviation. Statistical analyses were performed using XLSTAT 2025.1.1 Advanced software (Addinsoft, New York, NY, USA).

## 5. Conclusions

*H. moscheutos* is primarily known as an ornamental plant due to its large and striking flowers, which are among the most impressive within the genus, and its long blooming period, but it is also a valuable medicinal raw material.

The polyphenolic composition and in vitro bioactivity of an ethanolic extract from *H. moscheutos* flower petals were examined. The total phenolic content was approximately 22%, including phenolic acids (5.17 mg/g), flavanols (59.18 mg/g), flavonols (93.09 mg/g), and anthocyanins (62.08 mg/g).

Significant differences were observed in the effects of the extract on various groups of microorganisms. The extract exhibited predominantly weak to moderate antagonistic activity against multiple species within the genus *Staphylococcus*, with its efficacy strongly influenced by strain-specific characteristics. Although evidence suggests that the extract may serve as a potential alternative to certain antibiotics used against pathogenic strains of this genus, further research is necessary in this area. Other tested pathogenic strains, including *Enterococcus faecalis*, *Escherichia coli*, and *Salmonella* spp., were resistant to the extract even at the highest concentration applied (100 mg/mL). Additionally, no antifungal activity was detected against any of the tested yeast or filamentous fungal strains.

The extract did not show antagonistic activity against probiotic strains of *Bifidobacterium* spp. and most lactic acid bacteria. Furthermore, no selective stimulation of their growth over non-probiotic intestinal bacteria was observed, indicating that the extract lacks prebiotic activity in vitro. However, the extract was shown to enhance biofilm formation by both probiotic and pathogenic microbiota on abiotic (polystyrene) and biotic (porcine mucin and gelatin) surfaces. Notably, stimulation of biofilm formation in pathogenic *Staphylococcus* is undesirable, as it could lead to chronic, hard-to-treat infections, potentially limiting its pharmaceutical or medical applications. Concentrations above 25 mg/mL reduced the autoaggregation ability of lactic acid bacteria, bifidobacteria, and staphylococci. The mechanisms underlying these effects remain unknown and require further investigation at the molecular level. It should also be noted that the analyses were conducted only at the highest concentrations at which no growth inhibition of the tested microorganisms was observed. Therefore, the effects of other extract concentrations on these strains remain unclear.

In the Ames test, the extract exhibited low mutagenic activity within the concentration range of 1–10 mg/plate, corresponding to the highest doses used in this method. Additionally, it showed good in vitro anti-inflammatory activity—at concentrations of 250 and 500 µg/mL, it inhibited protein denaturation by 31.0% and 54.7%, respectively. Comparable values were obtained for sodium diclofenac, a nonsteroidal anti-inflammatory drug, at the same concentrations. The low genotoxicity at high doses and favorable anti-inflammatory activity make the ethanolic extract of swamp rose mallow a promising candidate for the development of new therapeutic strategies and functional foods. Nevertheless, further studies are warranted to fully ensure its safety.

## Figures and Tables

**Table 1:** Phenolic content in the freeze-dried ethanol extract of *H. moscheutos* petals.

Phenolic Compounds	Content, mg/g
Total phenolic acids	5.17 ± 0.14
Total flavanols (in this procyanidins)	59.18 ± 1.43 (53.63 ± 1.51)
Total flavonols	93.09 ± 0.57
Total anthocyanins	62.08 ± 0.51
Sum of phenolic compounds (HPLC)	219.52 ± 2.19
Total phenolics (F–C method) ^1^	216.39 ± 6.65

Results are expressed as mean ± standard deviation (*n* = 4). ^1^ mg gallic acid equivalent per gram of freeze-dried extract.

**Table 2:** Minimum inhibitory concentrations (MICs) of the extract against bacteria and growth inhibition zones at a concentration of 100 mg/mL, compared to positive controls.

Strain	Inhibition Zone, mm			MIC ^3^, mg/mL
	Extract ^1^	Positive Control ^2^		
	100 mg/mL	1	2	
**Probiotic bacteria**				
*Bifidobacterium breve* M-16V	–	23.0 ± 1.4 ^AMP2^	35.0 ± 1.4 ^C30^	>100.00
*Bifidobacterium infantis* 35624^®^	–	13.0 ± 1.4 ^AMP2^	30.0 ± 0.0 ^C30^	>100.00
*Lacticaseibacillus casei* ŁOCK 0900	21.0 ± 1.4	37.0 ± 1.4 ^AML10^	22.5 ± 0.7 ^AMP2^	12.5
*Lacticaseibacillus casei* ŁOCK 0908	–	25.0 ± 1.4 ^AML10^	15.0 ± 1.4 ^AMP2^	>100.00
*Lacticaseibacillus paracasei* ŁOCK 0919	13.0 ± 1.4	21.0 ± 1.4 ^AML10^	12.5 ± 0.7 ^AMP2^	100.00
*Lacticaseibacillus rhamnosus* GG	–	25.5 ± 0.7 ^AML10^	12.5 ± 0.7 ^AMP2^	>100.00
*Levilactobacillus brevis* ŁOCK 0944	–	23.0 ± 1.4 ^AML10^	12.0 ± 2.8 ^AMP2^	>100.00
*Limosilactobacillus reuteri* DSM 17938	–	13.0 ± 1.4 ^AML10^	– ^AMP2^	>100.00
**Other bacteria**				
*Enterococcus faecalis* ATCC 29212	–	18.5 ± 0.7 ^CN120^	8.0 ± 0.0 ^P10^	>100.00
*Enterococcus faecalis* ATCC 51299	–	6.0 ± 0.0 ^CN120^	7.5 ± 0.7 ^P10^	>100.00
*Escherichia coli* ATCC 11303	–	33.5 ± 0.7 ^C30^	26.5 ± 0.7 ^CN120^	>100.00
*Escherichia coli* ATCC 35218	–	10.0 ± 0.0 ^C30^	24.5 ± 0.7 ^CN120^	>100.00
*Salmonella* Choleraesuis ATCC 7001	–	27.5 ± 0.7 ^CN120^	– ^P10^	>100.00
*Salmonella* Enteritidis ATCC 13076	–	27.5 ± 0.7 ^CN120^	– ^P10^	>100.00
*Salmonella* Typhimurium ATCC 14028	–	24.5 ± 0.7 ^CN120^	– ^P10^	>100.00
*Staphylococcus aureus* ATCC 25923	12.0 ± 0.0	20.5 ± 0.7 ^FOX30^	33.5 ± 0.7 ^CIP5^	50.00
*Staphylococcus aureus* ATCC 27734	16.5 ± 0.7	18.5 ± 0.7 ^FOX30^	36.5 ± 0.7 ^CIP5^	100.00
*Staphylococcus aureus* ATCC 29733	15.5 ± 0.7	20.0 ± 0.0 ^FOX30^	30.0 ± 0.0 ^CIP5^	50.00
*Staphylococcus aureus* MW 040702	14.7 ± 0.6	16.5 ± 0.7 ^FOX30^	31.0 ± 0.0 ^CIP5^	25.00
*Staphylococcus capitis* MW 776357	31.3 ± 3.0	16.0 ± 0.0 ^FOX30^	38.5 ± 0.7 ^CIP5^	6.25
*Staphylococcus epidermidis* MW 040699	15.7 ± 3.2	17.0 ± 0.0 ^FOX30^	36.5 ± 0.7 ^CIP5^	100.00
*Staphylococcus epidermidis* MW 040700	–	27.0 ± 1.4 ^FOX30^	34.5 ± 0.7 ^CIP5^	>100.00
*Staphylococcus epidermidis* MW 040703	–	17.0 ± 1.4 ^FOX30^	31.5 ± 0.7 ^CIP5^	>100.00
*Staphylococcus haemolyticus* MW 040704	17.7 ± 0.6	15.0 ± 0.0 ^FOX30^	30.5 ± 0.7 ^CIP5^	12.50
*Staphylococcus haemolyticus* MW 776358	–	19.0 ± 1.4 ^FOX30^	38.5 ± 0.7 ^CIP5^	>100.00
*Staphylococcus saprophyticus* MW 040701	12.7 ± 0.6	12.0 ± 0.0 ^FOX30^	32.5 ± 0.7 ^CIP5^	25.00
*Staphylococcus warneri* MW 776360	–	20.0 ± 1.4 ^FOX30^	30.0 ± 0.0 ^CIP5^	>100.00
*Staphylococcus xylosus* MW 776359	–	19.0 ± 1.4 ^FOX30^	36.5 ± 0.7 ^CIP5^	>100.00

“–” No significant growth inhibition zone. ^1^ Diameter of wells: 10 mm. ^2^ Diameter of antibiotic discs: 6 mm. Antibiotics: AML10—amoxicillin (10 μg), AMP2—ampicillin (2 μg), FOX30—cefoxitin (30 μg), C30—chloramphenicol (30 μg), CIP5—ciprofloxacin (5 μg), CN120—gentamicin (120 μg), P10—penicillin (10 μg). ^3^ Minimum inhibitory concentration of the extract.

**Table 3:** Prebiotic properties of the ethanolic extract from *H. moscheutos* petals.

Probiotic Strain	Concentration, mg/mL	Prebiotic Index	
		ATCC 11303 ^1^	ATCC 35218 ^1^
*B. breve* M-16V	100.00	−0.12 ± 0.03	0.07 ± 0.02
*B. infantis* 35624^®^	100.00	−0.08 ± 0.04	0.11 ± 0.05
*L. casei* ŁOCK 0900	6.25	−0.32 ± 0.07	0.09 ± 0.10
*L. casei* ŁOCK 0908	100.00	−0.09 ± 0.12	0.10 ± 0.11
*L. paracasei* ŁOCK 0919	50.00	−0.03 ± 0.08	0.09 ± 0.02
*L. rhamnosus* GG	100.00	−0.15 ± 0.03	0.04 ± 0.02
*L. brevis* ŁOCK 0944	100.00	−0.12 ± 0.06	0.07 ± 0.05
*L. reuteri* DSM 17938	100.00	−0.15 ± 0.05	0.04 ± 0.04
*S. boulardii* CNCM I-745	100.00	−0.14 ± 0.01	0.05 ± 0.02

^1^ *E. coli* ATCC 11303 and *E. coli* ATCC 35218.

**Table 4:** Number of revertants per plate and mutagenicity index (MI) in *S.* Typhimurium strains TA98 and TA100 after treatment with various doses of *H. moscheutos* petal extract, with (+S9) and without (−S9) metabolic activation.

Treatment	Concentration, mg/Plate	Number of Revertants (CFU/Plate) and MI			
		TA98		TA100	
		−S9	+S9	−S9	+S9
Control	0.0	25 ± 4 ^B (c)^	27 ± 5 ^D (c)^	198 ± 11 ^B (b)^	241 ± 22 ^A (a, b)^
Extract	1.0	31 ± 5 (1.2) ^B (c)^	48 ± 1 (1.8) ^C (b)^	226 ± 23 (1.1) ^A, B (a b)^	219 ± 18 (0.9) ^A (a, b)^
Extract	5.0	57 ± 1 (2.3) ^A (b)^	105 ± 5 (3.8) ^A (a)^	262 ± 23 (1.3) ^A (a)^	210 ± 11 (0.9) ^A (a, b)^
Extract	10.0	59 ± 5 (2.4) ^A (b)^	91 ± 2 (3.3) ^B (a)^	230 ± 16 (1.2) ^A, B (a, b)^	220 ± 14 (0.9) ^A (a, b)^
Daunomycin	6.0 μg	575 ± 69	–	–	–
Sodium azide	1.5 μg	–	–	565 ± 52	–

“–” Not investigated. Number of analyzed plates: *n* = 3. ^A–C^ Statistically significant differences in the number of revertants compared to the control (without extract) in the +S9 or −S9 variant (ANOVA, *p* < 0.05). ^(a–c)^ Statistically significant differences between all treatments within the same strain (ANOVA, *p* < 0.05).

**Table 5:** In vitro anti-inflammatory properties of the ethanolic extract from *H. moscheutos* petals.

Treatment	Concentration, μg/mL	A660	% Inhibition
Control ^1^	–	0.848 ± 0.040	–
Extract	100	0.808 ± 0.020	4.73 ± 2.35 ^D^
Extract	250	0.585 ± 0.008	31.02 ± 0.90 ^B, C^
Extract	500	0.384 ± 0.033	54.70 ± 3.84 ^A^
DCF-Na ^2^	100	0.635 ± 0.004	25.15 ± 0.44 ^C^
DCF-Na	250	0.566 ± 0.043	33.26 ± 5.08 ^B^
DCF-Na	500	0.366 ± 0.065	56.82 ± 7.62 ^A^

^1^ distilled water (negative control), ^2^ DCF-Na—sodium diclofenac (positive control). ^A–D^ Statistically significant differences between treatments (ANOVA, *p* < 0.05; *n* = 3).

**Table 6:** Environmental isolates and their origin.

No.	Taxonomic Name	Strain ID ^1^	Origin
	**Dietary supplements and probiotic preparations**		
1	*Bifidobacterium breve*	M-16V	Bebilon^®^ Prosyneo™ HA 1 (NUTRICIA, Warsaw, Poland)
2	*Bifidobacterium infantis*	35624^®^	SYMBIOSYS Alflorex^®^ (BIOCODEX, Warsaw, Poland)
3	*Lacticaseibacillus rhamnosus*	ATCC 53103	Dicoflor^®^ 6 (Bayer, Warsaw, Poland)
4	*Limosilactobacillus reuteri*	DSM 17938	BioGaia Protectis^®^ Baby (BioGaia, Stockholm, Sweden)
5	*Saccharomyces boulardii*	CNCM I-745	Enterol^®^ (BioCodex, Gentilly, France)
	**Food and commercial starter cultures**		
6	*Penicillium candidum*	–	Lactoferm PC-1 Aroma-Tek^®^ (Biochem, Bologna, Italy)
7	*Saccharomyces cerevisiae*	–	Compressed yeast
8	*Staphylococcus capitis*	MW 776357 *	Radish sprouts
9	*Staphylococcus epidermidis*	MW 040699 *	Acai berry extract
10	*Staphylococcus epidermidis*	MW 040700 *	Acai berry extract
11	*Staphylococcus haemolyticus*	MW 776358 *	Fresh cow’s milk
12	*Staphylococcus warneri*	MW 776360 *	Radish sprouts
13	*Staphylococcus xylosus*	MW 776359 *	Fresh cow’s milk
	**Human skin**		
14	*Staphylococcus aureus*	MW 040702 *	Human foot skin
15	*Staphylococcus epidermidis*	MW 040703 *	Human hand skin
16	*Staphylococcus haemolyticus*	MW 040704 *	Human hand skin
17	*Staphylococcus saprophyticus*	MW 040701 *	Human foot skin

^1^ Identifier in a culture collection (ATCC, USA; CNCM, France; DSMZ, Germany), commercial designation, or * accession number in the NCBI database; “–” indicates no strain identifier.

## Data Availability

The data presented in this study are available upon request from the corresponding author. The data are not publicly available due to the volume and partial nature of the dataset.
